# Mutant Lines of Spring Wheat with Increased Iron, Zinc, and Micronutrients in Grains and Enhanced Bioavailability for Human Health

**DOI:** 10.1155/2019/9692053

**Published:** 2019-03-14

**Authors:** Saule Kenzhebayeva, Alfia Abekova, Saule Atabayeva, Gulzira Yernazarova, Nargul Omirbekova, Guoping Zhang, Svetlana Turasheva, Saltanat Asrandina, Fatma Sarsu, Yarong Wang

**Affiliations:** ^1^Department of Biotechnology, Kazakh National University, Named After Al-Farabi, Almaty, Kazakhstan; ^2^Kazakh Institute of Agricultural and Breeding, Almaty Region, Kazakhstan; ^3^Agronomy Department, Zhejiang University, Zijinggang Campus, China; ^4^The Plant Breeding and Genetics Section, Joint FAO/IAEA Division, International Atomic Energy Agency, Vienna, Austria

## Abstract

Deficiency of metals, primarily Fe and Zn, affects over half of the world's population. Human diets dominated by cereal products cause micronutrient malnutrition, which is common in many developing countries where populations depend heavily on staple grain crops such as wheat, maize, and rice. Biofortification is one of the most effective approaches to alleviate malnutrition. Genetically stable mutant spring wheat lines (M_7_ generation) produced via 100 or 200 Gy gamma treatments to broaden genetic variation for grain nutrients were analyzed for nutritionally important minerals (Ca, Fe, and Zn), their bioavailability, and grain protein content (GPC). Variation was 172.3–883.0 mg/kg for Ca, 40.9–89.0 mg/kg for Fe, and 22.2–89.6 mg/kg for Zn. In mutant lines, among the investigated minerals, the highest increases in concentrations were observed in Fe, Zn, and Ca when compared to the parental cultivar Zhenis. Some mutant lines, mostly in the 100 Gy-derived germplasm, had more than two-fold higher Fe, Zn, and Ca concentrations, lower phytic acid concentration (1.4–2.1-fold), and 6.5–7% higher grain protein content compared to the parent. Variation was detected for the molar ratios of Ca:Phy, Phy:Fe, and Phy:Zn (1.27–10.41, 1.40–5.32, and 1.78–11.78, respectively). The results of this study show how genetic variation generated through radiation can be useful to achieve nutrient biofortification of crops to overcome human malnutrition.

## 1. Introduction

Nutrient malnutrition represents one of the major health challenges worldwide and is characterized by an increasing number of people manifesting the condition in its varying forms [1–3]. Nearly 30% of humanity—infants, children, adolescents, adults, and older persons in the developing world—are currently suffering from one or more of the multiple forms of malnutrition [2]. Undernutrition and low dietary diversity are by far the biggest risk factors for this global disease and each country faces a serious public health challenge from malnutrition [3]. Iron (Fe) is a biologically essential element for humans, participating in a wide variety of metabolic processes, including oxygen transport, deoxyribonucleic acid (DNA) synthesis, and electron transport [4]. About 70% of the body's iron is bound to hemoglobin in red blood cells and to myoglobin in muscle cells [5]. The remaining iron is bound to other proteins (transferrin in blood or ferritin in bone marrow) or stored in other body tissues. Zinc (Zn) is another essential micronutrient for all living organisms, as it performs both catalytic and structural roles in a wide variety of proteins. One-tenth of the proteome, which is about 3000 human proteins, binds Zn [6, 7]. Disorders of Fe metabolism are among the most common nutritionally linked diseases of humans and encompass a broad spectrum of diseases with diverse clinical manifestations, ranging from anemia to iron overload, and possibly to neurodegenerative diseases. In terms of global scale and incidence of disease, iron deficiency ranks first and deficiency of zinc is third [8]. Many human disorders are related to Zn deficiency, such as impairment of development and growth, reduced immunity, and disorders of the nervous system [9].

Iron and zinc malnutrition, widely recognized as a major health problem, is predominantly caused by cereal-based diets that are deficient in micronutrients (Zn and Fe) and is prevalent in low-income and middle-income countries [10]. The consequences of malnutrition and nutrition-related diseases include impeded intrauterine growth, which affects 23.8% of all births per year, and protein-energy malnutrition (underweight) in 26.7% of children under-five worldwide, and over 60% of the world's people are Fe deficient and over 25% are Zn deficient [11]. Since Fe and Zn are often derived from the same nutrient-dense food sources in the human diet, lacking these foods generally results in a deficiency of both metals simultaneously.

The populations of the 22 developing countries of the world receive 43-78% of dietary Fe and 56-88% Zn from grains of C_3_-species and legumes [10]. Wheat (*Triticum aestivum* L.) is consumed as one of the major human foods and is a source of essential nutrients and protein for nutrition, with demand increasing due to the growing population [2]. Currently, wheat provides 28% of the world's edible dry matter and up to 60% of the daily calorie intake in developing countries [1, 12]. However, genetic gains in grain yield have not changed over recent years and nutritional value and particularly in the Fe and Zn grain protein content have been difficult to improve through traditional breeding [13, 14]. It is generally assumed that selective breeding narrowly focused on increasing yields has resulted in grains with a lower concentration of metals as result of a dilution effect [14, 15]. Furthermore, such a trend may worsen since some reports have shown that the edible portions of food crops grown in fields under elevated atmospheric CO_2_ have significantly decreased Fe contents by 4-10% [16].

Biofortification, or the process of genetic enhancement directly targeting the mineral status in the grains of staple food crops through breeding, is one of the most cost-effective and environmentally safe approaches to prevent and alleviate nutrient malnutrition in humans [17–20]. It was demonstrated, for example, that, with the inclusion of biofortified wheat in the human diet, Zn consumption was substantially higher relative to the nonbiofortified diet [21]. In addition, the biofortification of crops through breeding has multiplicative advantages such as long-term and sustainable means of delivering more micronutrients, maintaining improved nutritional status of malnourished people, and rise of the benefits of the initial investment [18].

Along with increasing the concentration of nutrients in food crops, a high bioavailability is also important for human nutrition [22]. Wheat foods are rich in antinutrients, especially phytic acid (Phy), which interferes with the absorption or utilization of nutrients in the digestive system [23]. In general, staple food crops and grains contain very low bioavailable Fe and Zn (i.e., about 5% of the total grain Fe and about 25% of the total Zn are bioavailable). To increase the Fe bioavailability from 5 to 20% it is roughly equivalent to increasing the total Fe four-fold [24]. It has been noted that it is genetically much easier to significantly improve the bioavailability of Fe and Zn in comparison to increasing their total concentration by this magnitude through conventional breeding [23]. Measuring mineral bioavailability in the human diet, their molar ratio with Phy has been widely used [4]. The regulation of Fe status in the human body is controlled through absorption, whereas Zn homeostasis is achieved through absorption but also gastrointestinal secretion and excretion of endogenous Zn [25].

Breeding for low phytic acid concentration is considered to be a reasonable objective to enhance the nutrient bioavailability of crop products. To reduce phytic acid, essential efforts have been made to mutagenize crops. Low phytate mutants (*lpa*) have been reported for several cereals using chemical and physical mutagenesis [26–29]. Successful breeding for yield-associated traits and increases in the nutritional value of cereal foods require genetic variation, which must be distinguishable from environmental effects. The genetic diversity of crops has decreased primarily as a consequence of breeding, including the repeated use of local germplasms and the adoption of breeding schemes that do not favour genetic recombination [27, 28]. Mutagenesis is a powerful tool to broaden genetic variation and has been used for yield increase but has been studied less for the improvement of grain nutritional value [28, 29]. To date, over 3275 mutant varieties in more than 220 plant species have been officially released worldwide [28]. Mutagenesis is especially valuable for inducing novel genetic variation in major crops that have limited genetic variability [29]. Importantly, mutant resources developed in crop breeding are not recognised as genetically modified organisms (GMO) and are freely distributed in all countries without restriction or public concern.

Medical studies have indicated that Phy inhibits Ca absorption, but its effect on Ca bioavailability seems to be less pronounced when compared to that of the bioavailability of Fe and particularly Zn [30]. This is possibly linked to the relatively high Ca concentration in cereal foods, the capability of the bacterial flora in the colon to dephosphorylate Phy, and the intake of Ca from the colon [31].

The nutritional value of crops is highly dependent on grain protein content (GPC), which has a significant impact on the end products [32]. Breeding for improvement of GPC is difficult due to the restricted range of GPC variation in available cultivars.

This study was undertaken with the following objectives: (1) to evaluate the variation in Ca, Fe, Zn, and Phy concentrations and grain protein content in spring wheat parental cv. Zhenis and advanced mutant lines (M_7_) produced via 100- and 200 Gy-gamma dose treatments; (2) to estimate the bioavailability of metallic nutrients; (3) to evaluate the correlations between thousand grain weight (TGW) and quality parameters. The comparison of mineral concentration in cv. Zhenis, 100- and 200 Gy-derived mutant lines, with recommended uptake from flour consumption was also determined.

## 2. Materials and Methods

### 2.1. Plant Material

Grains of the spring bread wheat (*Triticum aestivum *L.) cv. Zhenis were irradiated with 100 Gy and 200 Gy doses from a ^60^Co source at the Kazakh Nuclear Centre, Almaty. After irradiation, seeds were sown to raise M_1_ plants [33]. The M_1_ generation was grown in the experimental field of the Kazakh Institute of Agriculture and Breeding, Almaty district (43°15′N, 76°54′E, 550 m above sea level). Single spikes from each plant for the M_2_ generation were harvested, and the best lines were selected based on the yield of individual plants to continue to the M_7_ generation. The number of tillers and spikes per plant varied, but seeds were gathered only from a single main spike. Seeds from the best yielding mutant lines were individually selected in each generation. The selection criteria for these lines included grain weight per main spike (GWS) and per plant (GWP) and these were measured in the M_3_ and M_4_ generations (2011 and 2012), and compared to the values for the parental cv. Zhenis grown in the same trial conditions. In 2011, the parent had a mean grain weight per main spike of 1.20 ± 0.51 g and grain weight per plant of 1.85 ± 0.61 g. The threshold criteria for selection in the M_4_ generation were GWS > 1.4 g and GWP > 2.3 g for the mutant lines. The initial number of lines in the M_1_ generation was 2000 each for the 100 Gy and 200 Gy radiation doses. In the M_3_ generation, 61 lines (20%) were selected from the 100 Gy irradiation dose population and 48 lines (16%) were selected from the 200 Gy dose. The same numbers of lines for each radiation dose were selected for the M_4_-M_6_ generations. After harvesting the M_7_ plants, 23 lines and 8 lines from the original 100- and 200 Gy-derived germplasm were selected, respectively. The 100 Gy-dosed lines were numbered as follows: 6/9, 10/15, 11/6, 13/9, 15/1, 16/4, 17/7, 18/2, 20/10, 24/21, 26/5, 29/8, 36/13, 37/4, 39/2, 42/4, 43/43, 45/1, 47/2, 49/2, 52/1, 53/5, and 55/10; and 200 Gy-treated lines were numbered 57/4, 58/8, 59/2/, 61/2, 62/2, 63/2, 64/2, and 65/3. These mutant lines, selected from the two different levels of irradiation doses, were then used for further analysis for nutritional quality. Grain samples from each mutant line and the parent cv. Zhenis were sown together in a field trial and plants were grown in three replicates of three row plots, 2 m long x 1.2 m wide, and 20 cm between rows with 30 seeds planted per row for further evaluation. The trial was managed according to locally recommended agronomic practices. Applied fertilizers and their time of use, and soil conditions were as described earlier [33]. No additional fertilizer containing Fe and Zn or other inputs of these metals was applied. Ten randomly selected lines were taken for analysis (five samples per row). To record the three yield-associated traits, thousand-grain weight (TGW) was measured as the mean weight of three sets of 100 grains per line multiplied by 10.

### 2.2. Determination of Grain Protein Content

Grain protein content was determined with near-infrared reflectance (NIR) spectroscopy on whole grains (ZX50 Portable Grain 174 Analyzer, USA) using proprietary calibration software provided (Zeltex Hagerstown, 175 Ma USA). The measurements of 25 grains per line were repeated in triplicate.

### 2.3. Analysis of Grain Calcium, Iron and Zinc Concentrations

Grain samples (advanced M_7_ mutant lines and parent, cv. Zhenis) were washed with sodium dodecyl sulphate (0.1%), rinsed in deionized water, dried to a constant weight at 65-70°C, and then ground with a mixer mill (Retsch MM400 GmbH). The digestion and extraction of the sample (0.2 g) were as described [33]. Calcium, iron, and zinc concentrations were measured using flame atomic absorption spectroscopies Model NovAA350, AnalytikJena, Jena, Germany. Measurements of all minerals were checked against the certified reference values from the state standard samples LLC “HromLab”, Ca-7475-184 98, Fe-7254-96, and Zn-7256-96 diluted by 0.3% HNO_3_. Three extracts for analysis were performed.

### 2.4. Phytic Acid Extraction and Determination, and Molar Ratios of Phy:Metals and Ca:Phy

The extraction of Phy from the milled grain samples (0.3 g) was performed as described in [34]. A volume of 2.5 mL of the supernatant was treated with 2 mL 0.2% FeCl_3_, and the mixture was boiled for 30 min with further centrifugation after cooling. The residue was washed twice with deionized water. A total of 1.5 M NaOH were added to the precipitation, shaken and the solution was centrifuged. Three mL of 0.5 M HCl was added to the precipitation and then shaken until the precipitation dissolved. The solution was diluted to 25 mL to measure Fe residue by atomic absorption spectrophotometer (AAS, Shimadzu AA6300, Japan). Рhy sodium (Sigma St Louis, Missouri, USA) was used to test the Рhy recovery rate. The Рhy test results suggested that the recoveries were between 96 and 99%. The determination of Рhy was based on the precipitation of ferric phytate and measurement of Fe residue in the supernatant. The grain Рhy concentration was calculated by multiplying Fe content by a factor of 4.2. To calculate the molar ratios of Ca:Phy, Fe, and Zn, the concentrations of Рhy and the metals were converted into moles by dividing by their respective molar masses and atomic weights. The [Ca][Phy]/[Zn] (mol/kg) was also calculated.

### 2.5. Statistical Analysis

One-way ANOVA was used for comparisons; all data were evaluated in R 3.0.2 (R Core Development Team 2013). Simultaneous tests of general linear hypotheses and Dunnett's contrasts were used for multiple comparisons of the means. Summary data were reported as mean values ± standard deviations. Correlation coefficients between TGW and grain quality parameters and Probability* p-*values were calculated using GenStat software (10^th^ edition). A* p*-value of less than 0.05 was considered statistically significant.

## 3. Results

### 3.1. Variability in Ca, Fe, and Zn Concentrations in Grain of Spring Wheat M_7_ Mutant Lines and Parent

Significant differences in Ca, Fe, and Zn concentrations were found among the new spring wheat M7 mutant lines developed using dose radiation of 100 and 200 Gy and the parent cv. Zhenis.  Table 1 and Supplementary Table S1 show the means and ranges of the parameters. The CaC varied from 172.3-883.0 mg/kg in mutant lines (n = 93). Significantly enhanced CaC exceeded the parent by 1.23 to 2.47-fold, with the highest mean recorded in 100 Gy-treated lines identified in 15 M_7_ lines (48.4%).

Significant variation was also found for the microelements Fe and Zn between mutant lines derived from 100 and 200 Gy-irradiated lines, with means of 40.95-89.03 mg/kg and 22.2-89.6 mg/kg (n = 93), respectively. Significantly higher FeC and ZnC than the parent by 1.23 to 2.66- and 1.45-2.42-fold, respectively, were identified in 16 (51.6%) and 17 (54.8%) M_7_ lines. The GPC varied from 13.1 to 14.3% with a mean of 13.72 ± 0.26% (n = 93). Seventeen genotypes (54.8%), from the 100 Gy-dosed lines, had a significantly 6.5 to 7.6% higher GPC relative to the parent. The mutant lines exhibited wide variations in PhyC, from 1.17 to 2.66 mg/g (Table 1 and Supplementary Table S1). When compared to the parent, significantly lower PhyC by 1.25 to 2.02-fold was detected in 9 mutant lines (29%).

Analysis of variance (ANOVA) for differences in all nutrient concentrations among cv. Zhenis and mutant lines is shown in Table 2. These results revealed significant differences between the cv. Zhenis and 100 Gy- and 200 Gy-mutant lines for all traits except that of Phy:Zn for cv. Zhenis and the 200 Gy-treated lines (Table 2). However, the interactions between the 100 Gy- and 200 Gy-dosed lines were significant in terms of nutrient concentrations of FeC, GPC, and PhyC. For metals bioavailability, significant correlation among the 100 Gy- and 200 Gy-dosed lines was revealed for the Ca:Phy molar ratios. The radiation effect of 100 Gy was highest in GPC, indicating its increased efficiency to generate mutations in the genome associated with this trait. Traits such as FeC and therefore Phy:Fe showed greater variation in 200 Gy than 100 Gy treatments, showing that the most influence for their improvement was through the higher level of radiation.

Significant variations in the molar ratios of Phy:metals (Ca, Fe, and Zn) were also noted between the parent and mutant lines (Table 2). Variation was detected for the Ca:Phy, Phy:Fe, and Phy:Zn molar ratios, (1.27-10.41, 1.40-5.32, and 1.78-11.78, respectively) (Table 1 and Supplementary Table S1). The lowest means of these characteristics and therefore their highest bioavailability were present in the 100-Gy-derived mutant lines. The most significant noticeable variation in metal bioavailability between the parent and mutant lines was found for the Phy:Fe molar ratios, followed by Ca:Phy and Phy:Zn (Table 2). It was also observed that the 100 Gy-treated lines are significantly differed from the ones developed by the 200 Gy treatment in the Ca:Phy molar ratios (Table 2).

### 3.2. Correlations between Nutrient Concentration, Phytic Acid, and Thousand-Grain Weight

To examine the relationships between nutrient concentrations, Phy content and yield, a correlation analysis (R^2^) was conducted; data are presented in Table 3. In the parent, several traits showed consistent correlations between each other. For instance, among the metals, a highly significant relationship was detected between CaC and FeC and to a lesser degree between FeC and ZnC. The correlations between PhyC and the metal concentrations were highly significant for ZnC, CaC, and FeC.

Generally, the correlations between the investigated traits were found to be lower for the 100 Gy- and 200 Gy-derived lines than the cv. Zhenis. There was a significant and positive association between FeC and ZnC (r^2^ = 0.11-0.35, respectively, p<0.01) (Table 3). We revealed significant and positive associations between FeC and TGW in the 100 Gy- and 200 Gy-derived lines, but not with the parent, with the highest mean in the 200 Gy-dosed lines. The distinctive significant feature in the 200 Gy-dosed lines was the correlation between PhyC with TGW, GPC, and also FeC, but not with ZnC. The relations between Phy:microelements and Ca:Phy molar ratios and TGW and GPC were also analysed (Table 4). In the parent cv. Zhenis, there was only a significant relation between TGW and Phy:Zn. The 100 Gy- and 200 Gy-derived lines presented significant correlations between TGW and the Phy:Fe molar ratio, with the mean more than two times higher in 200 Gy-dosed lines. These results indicate highly possible simultaneous improvement of Fe bioavailability with the spring wheat productivity component. A significant relationship between the Phy:Zn molar ratios and GPC was only detected in the 200 Gy-treated mutant lines.

### 3.3. Estimated Nutrient Bioavailability in Wheat Flours

To determine whether the highest means of mineral concentrations from the parent and mutant lines provided the required daily intake of Fe, Zn, and Ca, we calculated the ratio of grain mineral concentration from 200 g flour consumption to the percentage of the recommended uptake (Table 5). These calculations were based on the statistics from the FAO [35], where the mean consumption of wheat flour is about 200 g per person per day, and on the values for the recommended intake for adults according to the DGE (German Nutrition Society) [36]. The results obtained in this study indicated that, in the case of the parent cv. Zhenis, Fe deficiency was highly manifested. The highest concentration of these minerals from the mutant lines that produced whole grain flour was revealed to provide 1.28-1.32-fold more than the required daily intake of Fe, Zn, and Mg. The Ca concentrations in the grain of the parent and mutant lines supplied were 5.05-12.5 and 1.46-2.44-fold higher, respectively, when compared to the required daily consumption of these minerals.

## 4. Discussion

This study reported the production of genetically stable advanced (M_7_) mutant lines of spring wheat derived from 100 Gy and 200 Gy irradiation treatment showing exceptionally high concentrations of nutritionally important nutrients (Ca, Fe, Zn, and GPC) with accompanying analysis of their bioavailability (Table 1 and Supplementary Table S1). The variation in grain nutrient content was 172.3-883.0 mg/kg for CaC, 31.1-89.0 mg/kg for FeC, and 22.2–89.6 mg/kg for ZnC. Of the minerals investigated, the greatest increase in concentration in the mutant lines compared to the parent was found in FeC, followed by CaC and ZnC with means of 2.66, 2.47, and 2.42, respectively. 17 genotypes (54.8%), of the 100 Gy-derived lines had a significant 6.5 to 7.6% higher GPC relative to the parent.

The concentrations of minerals in the wheat mutant lines exceeded those already reported earlier for hexaploid wheat by 2.35-2.96-fold for FeC [14, 15, 37–39]. Although environmental factors can influence grain metal concentrations, in this work, all the mutant lines and the parent were grown under the same field conditions and were treated equally, with no specific fertilizer supplementation or other inputs of these metals. Studies that compared historical and modern wheat cultivars for the evaluation of grain yield and concentration of Ca, Cu, Fe, Zn, Mg, Mn, P, and Se reported that, over time, the concentrations of all minerals, except Ca, decreased, while grain yield increased [14, 15]. Therefore, this situation suggests that a greater consumption of wheat bread from modern cultivars is required to achieve the same percentage of recommended dietary allowance levels as was provided by older cultivars with lower yield.

Micronutrient intake from wheat is essentially determined by the amount available for human absorption. High micronutrient bioavailability can be achieved by the reduction of antinutritional agents. The Phy level is considered to be one of the most important causative factors limiting metal bioavailability through chelation [17, 20, 22]. However, it was recently suggested that sulphur containing peptides rather than Phy bind to Zn in barley [40]. The function of Phy is as a phosphorus and energy store, and a source of cations and myoinositol could be improved by decreasing PhyC.

Breeding for low PhyC is a reasonable objective to enhance nutrient bioavailability in grain. To reduce PhyC content, essential efforts have been made to mutagenize crops. Low phytate cereal mutants (*lpa*) have been reported using chemical and physical mutagenesis [26, 41, 42]. In wheat, the* lpa* mutant was isolated by chemical mutagenesis [43]. At the same time,* lpa* mutations in several crops usually lead to pleiotropic effects on plant and seed performance, such as reduced germination and emergence rate, lower seed filling, and susceptibility to stress [42]. Our study showed that low Phy wheat lines generated from cv. Zhenis by 100 Gy and 200 Gy radiation did not display any differences in seed viability or shoot and root growth when compared with the parent (data not shown).

The variation found for PhyC ranged from 1.17 to 2.66 mg/g (2.3-fold variation) in all of the mutant lines (Table 1 and Supplementary Table S1) with the lowest PhyC recorded in the 100 Gy-derived line numbered 24(21), which was two times lower relative to that of the parent. There were significant differences between the 100 Gy- and the 200 Gy-derived mutant lines (Table 2). In addition, a search for low Phy lines among the M_7_ mutant lines generated by 100 Gy on the genetic background of cv. Erythrospermum 35 allowed us to identify the lowest PhyC, which was 3.5-fold lower than the parent [33].

Studies of natural wheat variation revealed huge differences in PhyC content. The range of variation found was of 5.9-45.4 mg/g [44–47]. However, it is possible that these findings are inconsistent due to differences in the methodology employed for determination of Phy levels, as indicated by Gibson et al. [48], stressing that selection of the most appropriate method for Phy analysis is critical.

The potential bioavailability of nutrients for human consumption is estimated by Phy:metal molar ratios, or* vice versa* for the microelements. In general, low molar ratio means high mineral bioavailability and the same conversely. In the current study, significant variability for the Ca:Phy, Phy:Fe, and Phy:Zn molar ratios between the parent and mutant lines was detected (Table 1 and Supplementary Table S1). Among them, the most pronounced variation was for Ca:Phy (more than 7-fold) and for Phy:Zn (around 7-fold) in the 100 Gy-dosed mutant lines. A high level of variation for Phy:Fe (1.40-5.32) with a mean of about a 4-fold difference was also detected. Therefore, it seems clear that the genetic variability available in mutant lines of spring wheat is enough to warrant their use as resources in breeding. To significantly increase Fe and Zn absorption Phy:Fe molar ratios was estimated at <1 or preferably <0.4 [49] and for the Phy:Zn molar ratio, <5 was considered high Zn bioavailability, corresponding to approximately 50% of the total Zn [50]. Phy:Fe ratios with a reported range similar to that obtained in the current study (1.96-3.86) were reported in 12 bread wheat varieties [51]. Higher Phy:Fe molar ratios have been reported in the literature, such as means of around 12 in two bread wheat cultivars [52] and of 15.5-31.3 in a set of nine bread wheat varieties [53]. Concerning the Phy:Zn molar ratio, a range of 23.9-41.4 was found in 65 bread wheat varieties from Pakistan [54], and higher means of 29-178 were revealed in bread wheat [55].

In medical studies, the molar ratio of [Ca][Phy]/[Zn] is a better indicator of Zn bioavailability, as Ca strengthens the effect of Phy on Zn absorption due to the existence of a kinetic synergism between Ca and Zn ions that results in the formation of a more insoluble Ca:Zn:Phy complex when compared to the Phy complexes formed by either alone. Therefore, the [Ca][Phytate]/[Zn] molar ratio is a better index for predicting Zn bioavailability than the Phy:Zn ratio because of this Ca-Phy interaction.

A [Ca][Phy]/[Zn] molar ratio greater than 0.5 mol/kg may reduce Zn bioavailability [56]. The considerable variation found for the [Ca][Phytate]/[Zn] molar ratio (0.21-3.32) in the current study in mutant lines (Table 1) was below the critical level. This means that there was a 11.0-14.3-fold variation across the lines, and the ones with the lowest values for [Ca][Phy]/[Zn] fall in the category of high Zn bioavailability according to the designation suggested by the authors [56].

## 5. Conclusions

The data generated in the present study has shown considerable variation in nutrients (Ca, Fe, Zn, and GPC) and Phy in new spring wheat mutant lines (M_7_) that were derived from 100 and 200 Gy treatment of the parent cv. Zhenis. The results showed that among these mutant resources a great number of lines (8 genotypes) have a significantly higher Phy:Zn and Ca concentration than that of the parent. Of these, 4 lines also recorded simultaneously high bioavailability of Zn, Fe, and Ca. In addition to these valuable characteristics of grain nutritional quality, the line 6/9 also recorded a high GPC. The only correlation between TGW and nutrient content was found for concentrations of Fe in the mutant lines; in addition, Ca and Phy contents were correlated, and a significant correlation existed between GPC and the concentration of Phy. Thus, consumption of whole wheat bread produced from these new mutant lines could contribute a higher percentage of recommended dietary allowance levels of these essential nutrients. The promising mutant lines identified could be useful to generate mutant varieties with appropriate levels of bioavailable metals, which can lead to the development of variety-based products rich in the desired minerals to overcome deficiencies in human intake.

## Figures and Tables

**Table 1:** Comparison between trait means and ranges for spring wheat cv. Zhenis (parent) and M_7_ derived from 100 Gy- and 200 Gy-irradiated mutant lines. Data are shown as mean and range (n=93).

Trait	cv. Zhenis		100 Gy-dosed lines		200 Gy-dosed lines	

	Mean	Range	Mean	Range	Mean	Range

TGW (g)	40.75	40.35–41.45	42.46	36.25–57.4	41.15	33.25–52.15

GPC, %	13.00	13.0–13.1	13.85	13.6–14.3	13.34	13.2–13.7

CaC (mg/kg)	357.07	350.6–365.1	568.13	172.30–883.0	530.80	203.4–856.5

FeC (mg/kg)	33.20	31.10–35.10	57.20	40.95–88.83	65.09	44.57–89.03

ZnC (mg/kg)	36.1	33.1–39.3	56.03	22.2–89.60	56.40	22.2–88.10

PhyC (mg/g)	2.59	2.58–2.63	2.23	1.17–2.66	2.47	2.02–2.65

Ca:Phy	2.26	2.24–2.28	4.34	1.39–10.41	3.57	1.27–5.95

Phy:Fe	6.65	6.21–7.19	3.49	1.40–5.32	3.39	2.18–4.90

Phy:Zn	7.16	6.66–7.71	4.48	1.78–11.78	5.59	2.60–11.19

[Ca][Phy]/[Zn] (mol/kg)	0.97	0.92–1.05	1.01	0.21–3.01	1.15	0.30–3.32

*Note.* Each line was analysed by three replicates. TGW was measured for 15 replicates.

**Table 2:** Comparison of Ca, Fe, Zn, and phytic acid concentrations and Phy:microelements and Ca:Phy molar ratios of advanced M_7_ mutant lines and parent grain of spring wheat cv. Zhenis.

Source of variation	cv. Zhenis x 100 Gy-dosed lines	cv. Zhenis x 200 Gy-dosed lines	100 Gy- x 200 Gy-dosed lines
Df	83	38	92

CaC	23.13 *∗∗∗*	28.89*∗∗∗*	0.74

FeC	64.97*∗∗∗*	203.83*∗∗∗*	7.15*∗*

ZnC	21.87*∗∗∗*	26.98*∗∗∗*	0.01

GPC	647.78*∗∗∗*	149.29*∗∗∗*	244.41*∗∗∗*

PhyC	16.20*∗∗∗*	19.62*∗*	9.79*∗∗∗*

Ca:Phy	27.18*∗∗∗*	36.50*∗∗∗*	4.49*∗*

Phy Fe	169.05*∗∗∗*	468.55*∗∗∗*	0.20

Phy:Zn	29.50*∗∗∗*	10.08	4.29

*Note.* Data are presented as a percentage of the total sum from ANOVA analysis. The lines were significantly different from the parental line. Asterisks, *∗*, *∗∗*, and *∗∗∗*, denote significance at the P<0.05, 0.01, and 0.001 level, respectively.

**Table 3:** R^2^ correlation coefficients between nutrient concentration, phytic acid, and thousand grains weight, with *p-*values denoted by asterisks.

Parameters	TGW	GPC	FeC	ZnC	CaC
*cv. Zhenis*					

GPC, %	0.024				

FeC (mg/kg)	0.002	0.000			

ZnC (mg/kg)	0.622*∗∗∗*	0.017	0.34*∗*		

CaC (mg/kg)	0.011	0.000	0.98*∗∗∗*	0.052	

PhyC (mg/g)	0.454*∗∗*	0.0128	0.504*∗∗*	0.972*∗∗∗*	0.646*∗∗∗*

*100 Gy-derived lines*					

GPC, %	0.01				

FeC (mg/kg)	0.081*∗*	0.014			

ZnC (mg/kg)	0.084*∗*	0.000	0.11*∗∗*		

CaC (mg/kg)	0.005	0.025	0.045	0.063*∗*	

PhyC (mg/g)	0.092*∗∗*	0.012	0.080*∗*	0.043	0.021

*200 Gy-derived lines*					

GPC, %	0.005				

FeC (mg/kg)	0.220*∗*	0.011			

ZnC (mg/kg)	0.009	0.012	0.35*∗∗*		

CaC (mg/kg)	0.03	0.014	0.036	0.004	

PhyC (mg/g)	0.323*∗∗*	0.254*∗*	0.273*∗∗*	0.027	0.011

*Note.* The lines were significantly different from the parent line. Each line was analyzed by three replicates. Asterisks, *∗*, *∗∗*, and *∗∗∗*, denote significance at the P<0.05, 0.01, and 0.001 probability level, respectively.

**Table 4:** Association between Phy:nutrients and Phy:Ca molar ratios and thousand grain weight and grain protein content for cv. Zhenis spring wheat and advanced mutant lines (100 Gy- and 200 Gy-derived).

Trait	Phy:Fe	Phy:Zn	Ca:Phy
*cv. Zhenis*			

TGW, g	0.007	0.759*∗∗∗*	0.126

GPC, %	0.00	0.013	0.002

*100 Gy-derived lines*			

TGW, g	0.086*∗*	0.02	0.03

GPC, %	0.025	0.005	0.00

*200 Gy-derived lines*			

TGW, g	0.222*∗*	0.033	0.003

GPC, %	0.0012	0.141*∗*	0.014

*Note.* The lines were significantly different from the parent line. Each line was analyzed by three replicates. Asterisks, *∗*, *∗∗*, and *∗∗∗*, denote significance at the p<0.05, 0.01, and 0.001 probability level, respectively.

**Table 5:** Comparison of mineral concentration in cv. Zhenis, 100 Gy-, and 200 Gy-derived lines with the recommended uptake of minerals (mg/day) according to DCE, percentage of recommended uptake from flour consumption of 200 g/person/day (mg/day), mg minerals from 200 g flour consumption obtained from max values of genotypes, and ratio of grain mineral concentration from percentage of recommended uptake.

Mine-ral concentration (mg/kg)	Genotypes		Recommended uptake of minerals	Percentage of recommended uptake	mg minerals from 200 g flour consumption			Minerals mg from percentage of recommended uptake	Ratio of grain minerals concentration		
	cv. Zhenis	100 Gy- derived lines			cv. Zhenis	100 Gy-derived lines	200 Gy-derived lines		cv. Zhenis	100 Gy-derived lines	200 Gy-derived lines

Fe	31.3	88.8	10	76	6.3	17.8	17.8	13.5	0.35	1.32	1.32

Zn	36.1	89.6	10	78	7.2	17.9	17.62	14.0	0.40	1.28	1.26

Ca	357.1	883.0	1000	8	71.4	176.6	171.3	14.1	5.05	12.50	12.12

## Data Availability

The data used to support the findings of this study are available from the corresponding author upon request.

## Supplementary Materials

Supplementary Materials Brief description of Supplementary Table S1: variability of grain traits such as concentrations of Fe, Zn, Ca and phytic acid (Phy), grain protein content (GPC), molar ratios of phytic acid to Fe (Phy:Fe), Zn (Phy:Zn), Ca (Ca:Phy), and grain thousand weight (TGW) in spring wheat parental cv. Zhenis and 100- and 200-Gy derived M7 mutant lines. The traits were measured in triplicates in parent and each mutant line.
