# Supplementary material for: Mutant Lines of Spring Wheat with Increased Iron, Zinc, and Micronutrients in Grains and Enhanced Bioavailability for Human Health
Source: Biomed Res Int. 2019 Mar 14;2019:9692053. doi: 10.1155/2019/9692053 (PMC6437721; doi:10.1155/2019/9692053)
Supplement: Supplementary Materials — Brief description of Supplementary Table S1: variability of grain traits such as concentrations of Fe, Zn, Ca and phytic acid (Phy), grain protein content (GPC), molar ratios of phytic acid to Fe (Phy:Fe), Zn (Phy:Zn), Ca (Ca:Phy), and grain thousand weight (TGW) in spring wheat parental cv. Zhenis and 100- and 200-Gy derived M7 mutant lines. The traits were measured in triplicates in parent and each mutant line. [file 9692053.f1.pdf]

Supplementary Table S1. Variability of grain traits: concentraions of Fe, Zn, Ca and phytic acid (Phy), gra content (GPC), molar rations of phytic acid to Fe (Phy:Fe), Zn (Phy:Zn) and Ca:Phy molar ratio and 1000 g weight (TGW) in spring wheat parental cv. Zhenis and 100- and 200-Gy derived M_7 mutant lines.
The traits were measured in triplicates in each mutant line.

Genotype	Fe, mg/kg	Zn, mg/kg	Ca, mg/kg	GPC,%	Phy, mg/g	Phy:Fe, mol	Phy:Zn mol	Ca:Phy mol
Zhenis-1	35,10	35,9	350,6	13,0	2,581	6,21	7,10	2,24
Zhenis-2	31,10	39,3	365,1	13,0	2,625	7,19	6,66	2,28
Zhenis-2	33,24	33,1	355,5	12,9	2,562	6,56	7,71	2,27
39/2-1	65,87	42,7	697,2	13,8	2,477	3,22	5,21	5,12
39/2-2	64,90	45	617,2	14,0	2,547	3,36	4,94	4,53
39/2-3	64,73	49,3	683,4	13,9	1,725	2,25	4,51	5,01
13/9-1	48,03	25,3	563,8	13,9	2,504	4,42	9,82	3,70
13/9-2	44,93	22,2	668,9	13,8	2,660	4,98	11,78	4,17
13/9-3	44,04	23,1	638,8	14,0	2,611	5,08	11,32	3,99
29/8-1	44,51	52,7	883,0	14,0	2,589	4,90	4,84	5,65
29/8-2	42,02	52,3	879,9	13,9	2,568	5,19	4,88	5,63
29/8)-3	40,98	52,4	872,0	13,9	2,576	5,32	4,87	5,58
10/15-1	51,66	35,1	585,8	13,9	2,607	4,22	7,27	3,75
10/15-2	51,58	35,3	554,5	13,9	2,627	4,34	7,41	3,46
10/15-3	53,82	38,1	519,1	13,9	2,607	4,05	6,69	3,32
42/4-1	70,56	66,5	449,3	13,9	2,604	3,09	3,84	2,87
42/4-2	75,59	61,2	443,6	14,1	2,472	2,74	3,95	2,99
42/4-3	72,65	63,6	426,1	13,8	2,443	2,85	3,80	2,87
36/13-1	50,68	53,8	407,8	13,9	2,350	3,97	4,38	2,83
36/13-2	53,76	54,8	404,8	14,0	2,552	4,06	4,65	2,59
36/13-3	59,73	54,8	437,9	13,9	2,523	3,56	4,54	2,88
43/43-1	47,03	65,3	873,3	14,0	2,475	4,40	3,71	5,89
43/43--2	47,03	68,1	865,2	14,0	2,587	4,64	3,75	5,54
43/43-3	45,82	66,5	861,3	14,0	2,522	4,64	3,74	5,66
16/4-1	49,49	61,1	190,8	13,9	1,958	3,39	3,21	1,59
16/4-2	47,14	63,6	172,3	13,9	1,665	2,96	3,19	1,39
16/4-3	48,84	63,8	191,2	13,8	2,612	4,58	3,28	1,49
37/4-1	52,81	54,8	701,8	13,9	2,520	4,02	4,54	4,61
37/4-2	51,13	48,8	739,0	14,0	2,599	4,26	5,23	4,73
37/4-3	52,27	47,9	710,5	14,1	2,484	4,06	5,19	4,67
18/2-1	47,90	45,8	778,7	13,9	2,517	4,43	5,43	5,88
18/2-2	47,93	21,8	711,8	14,1	2,596	4,55	6,10	5,24
18/2-3	48,46	32,1	776,2	13,9	2,577	4,50	7,95	5,71
49/2-1	42,76	67,9	345,8	13,8	2,513	4,97	3,66	2,27
49/2-2	40,99	68,4	355,9	13,9	2,591	5,32	3,73	2,28
49/2-3	45,74	61,8	355,5	13,9	2,575	4,77	4,13	2,27
52/10-1	62,10	72,1	619,7	13,7	1,882	2,61	2,72	5,15
52/10-2	63,08	79,6	631,7	13,7	2,335	3,10	2,79	4,64
52/10-3	62,31	77,8	627,4	13,8	2,201	2,96	2,77	4,74
6/9-1	50,30	67,9	733,0	13,8	2,288	3,89	3,37	5,23
6/9-2	55,34	68,4	764,4	13,9	2,215	3,43	3,25	5,61
6/9-3	58,66	61,8	767,2	14,2	2,164	3,14	3,49	5,80
53/5-1	63,28	82,1	524,6	13,7	2,234	3,00	2,71	3,85
53/5-2	64,21	89,6	534,1	13,8	2,314	3,05	2,55	3,81
53/5-3	66,51	87,8	519,1	13,7	2,180	2,77	2,46	3,92
24/21-1	74,98	61	442,1	13,7	1,319	1,49	2,14	5,52
24/21-2	71,22	64,1	431,6	13,8	1,759	2,12	2,14	5,13
24/21-3	72,02	66,3	421,5	13,8	0,768	0,93	1,78	5,84
45/1-1	44,23	86,9	414,2	13,6	2,111	4,04	2,41	3,23

45/1-2	41,16	89,5	420,6	13,6	2,192	4,48	2,41	3,18
45/1-3	41,52	85,3	437,4	13,7	2,161	4,44	2,53	3,31
26/5-1	83,56	22,2	533,3	13,6	1,865	1,87	8,25	4,75
26/5-2	86,52	23,1	521,5	13,9	2,190	2,13	9,34	3,94
26/5-3	88,33	24,8	561,3	13,9	1,836	1,77	7,38	5,00
11/6-1	55,24	40,8	708,1	13,7	2,018	3,14	4,97	5,70
11/6-2	51,82	40,9	702,3	13,8	2,140	3,45	5,12	5,48
11/6-3	53,27	40,7	736,4	14,0	1,337	2,10	3,21	9,19
47/2-1	49,03	41,8	288,1	13,8	1,955	3,42	4,69	2,40
47/2-2	48,31	42,1	298,4	13,9	1,845	3,24	4,35	2,66
47/2-3	48,31	49,6	291,5	14,0	1,640	2,89	3,30	2,91
20/10-1	52,14	40,2	832,5	13,9	1,755	2,89	4,07	8,31
20/10-2	50,37	44,1	811,8	13,9	1,634	2,77	3,71	8,10
20/10-3	55,77	48,3	876,2	13,8	1,289	2,00	2,84	10,41
17/7-1	85,48	68,9	354,5	13,8	1,261	1,24	2,37	3,54
17/7-2	86,09	69,5	365,6	13,8	2,456	2,40	3,20	2,68
17/7-3	87,03	65,3	381,0	13,7	2,458	2,38	3,41	2,80
55/10-1	55,87	52,2	562,4	13,6	2,480	3,80	4,76	3,69
55/10-2	53,52	53,1	571,5	13,8	2,615	4,18	4,93	3,57
55/10-3	58,57	52,7	572,5	13,9	2,393	3,44	4,47	3,97
15/1-1	68,42	92,3	538,8	13,7	2,146	2,61	2,78	4,20
15/1-2	62,52	72,4	556,4	13,6	2,534	3,40	3,43	3,65
15/1-3	60,51	75,1	486,1	13,6	2,456	3,42	3,22	3,43
65/3-1	44,57	79,6	844,3	13,6	2,513	4,77	3,12	5,54
65/3-2	47,35	85,3	856,5	13,5	2,569	4,60	2,99	5,48
65/3-3	48,37	88,1	834,5	13,6	2,335	4,04	2,60	5,95
58/8-1	66,81	86,5	439,5	13,5	2,482	3,18	2,87	2,89
58/8-2	63,81	81,2	442,7	13,6	2,207	2,89	2,66	3,35
58/8-3	67,85	83,6	435,1	13,5	2,377	2,97	2,82	3,02
63/2-1	87,19	23,8	454,5	13,4	2,276	2,18	9,34	3,34
63/2-2	89,03	24,8	445,1	13,6	2,382	2,26	9,49	3,09
63/2-3	88,81	24,8	475,4	13,4	2,399	2,27	9,49	3,29
64/-2-1	78,93	77,8	566,1	13,3	2,016	2,20	2,61	4,55
64/2-2	77,28	60,2	545,1	13,4	2,393	2,60	3,91	3,78
64/2-3	72,51	74,1	564,5	13,5	2,231	2,62	3,00	4,14
57/4-1	47,26	68,3	203,4	13,2	2,641	4,73	3,83	1,27
57/4-2	47,99	68,9	239,0	13,4	2,655	4,66	3,80	1,49
57/4-3	45,61	69,5	210,5	13,2	2,626	4,90	3,76	1,31
61/2-1	61,51	25,3	764,8	13,3	2,615	3,45	10,34	4,77
61/2-2	65,64	22,2	777,8	13,2	2,525	3,24	11,19	5,11
61/2-3	69,61	23,1	776,2	13,2	2,553	3,13	11,04	4,97
59/2-1	75,88	32,26	322,6	13,1	2,595	2,87	7,91	2,06
59/2-2	72,16	33,41	334,1	13,3	2,535	2,94	7,44	2,19
59/2-3	75,26	32,56	325,6	13,4	2,530	2,82	7,63	2,14
62/2-1	53,88	62,26	622,5	13,1	2,621	4,15	4,20	3,88
62/2-2	57,92	63,41	634,1	13,2	2,606	3,76	4,02	4,06
62/2-3	57,03	62,54	625,4	13,1	2,638	3,92	4,18	3,90

ain protein
rain

[Ca][Phy]/[Zn] (mol/kg)	TGW, g
0,95	40,35
0,93	40,45
1,05	45,45
1,39	41,45
1,16	42,75
1,18	38,00
2,11	41,35
3,01	39,30
2,76	42,25
1,63	51,45
1,64	44,80
1,62	50,35
1,62	47,95
1,57	48,45
1,33	44,50
0,66	43,25
0,67	42,75
0,62	46,50
0,68	52,95
0,72	54,00
0,76	57,40
1,24	42,90
1,24	43,05
1,23	43,30
0,23	39,10
0,21	40,70
0,24	42,25
1,21	39,40
1,47	37,60
1,41	41,85
1,85	42,05
1,91	41,65
2,71	41,90
0,48	45,95
0,51	45,30
0,56	45,55
0,64	37,45
0,67	38,65
0,66	43,30
0,94	42,65
0,95	46,60
1,02	42,30
0,54	41,85
0,52	42,10
0,49	43,10
0,36	44,75
0,35	43,80
0,29	43,00
0,38	43,05

0,39	38,50
0,42	44,20
1,68	33,25
1,86	36,75
1,58	36,85
1,34	42,10
1,37	37,25
0,90	42,40
0,52	42,60
0,50	43,35
0,37	43,05
1,29	41,30
1,15	41,65
0,95	41,80
0,32	41,30
0,45	44,70
0,50	42,00
1,02	41,15
1,07	41,80
0,98	39,45
0,57	46,95
0,73	41,65
0,63	42,30
1,01	43,25
0,98	41,90
0,83	42,85
0,48	42,65
0,45	42,55
0,47	42,70
1,62	35,30
1,61	36,50
1,72	34,10
0,56	35,10
0,81	34,15
0,65	32,25
0,30	40,65
0,35	45,45
0,30	41,40
3,02	45,20
3,32	44,10
3,27	52,15
0,97	43,30
0,95	42,05
0,95	44,95
1,00	44,75
0,97	41,40
1,00	40,30
